# Maximizing Insights from Longitudinal Epigenetic Age Data: Simulations, Applications, and Practical Guidance

**DOI:** 10.21203/rs.3.rs-4482915/v1

**Published:** 2024-06-20

**Authors:** Anna Großbach, Matthew J. Suderman, Anke Hüls, Alexandre A. Lussier, Andrew D.A.C. Smith, Esther Walton, Erin C. Dunn, Andrew J. Simpkin

**Affiliations:** (1) School of Mathematical and Statistical Sciences, University of Galway, Ireland;; (2) The SFI Centre for Research Training in Genomics Data Science, Ireland;; (3) MRC Integrative Epidemiology Unit, Population Health Sciences, Bristol Medical School, University of Bristol, Bristol, UK;; (4) Department of Epidemiology, Rollins School of Public Health, Emory University, Atlanta, GA, United States;; (5) Gangarosa Department of Environmental Health, Rollins School of Public Health, Emory University, Atlanta, GA, United States;; (6) Department of Biostatistics and Bioinformatics, Rollins School of Public Health, Emory University, Atlanta, GA, United States;; (7) Psychiatric and Neurodevelopmental Genetics Unit, Center for Genomic Medicine, Massachusetts General Hospital, Boston, MA, USA;; (8) Department of Psychiatry, Harvard Medical School, Boston, MA, USA;; (9) Stanley Center for Psychiatric Research, The Broad Institute of Harvard and MIT, Cambridge, MA, USA;; (10) Mathematics and Statistics Research Group, University of the West of England, Bristol, UK; (11) Department of Psychology, University of Bath, Bath, UK;

**Keywords:** epigenetic age, longitudinal studies, ALSPAC, accelerated aging, DNA methylation

## Abstract

**Background:**

Epigenetic Age (EA) is an age estimate, developed using DNA methylation (DNAm) states of selected CpG sites across the genome. Although EA and chronological age are highly correlated, EA may not increase uniformly with time. Departures, known as epigenetic age acceleration (EAA), are common and have been linked to various traits and future disease risk. Limited by available data, most studies investigating these relationships have been cross-sectional - using a single EA measurement. However, the recent growth in longitudinal DNAm studies has led to analyses of associations with EA over time. These studies differ in (i) their choice of model; (ii) the primary outcome (EA vs. EAA); and (iii) in their use of chronological age or age-independent time variables to account for the temporal dynamic. We evaluated the robustness of each approach using simulations and tested our results in two real-world examples, using biological sex and birthweight as predictors of longitudinal EA.

**Results:**

Our simulations showed most accurate effect sizes in a linear mixed model or generalized estimating equation, using chronological age as the time variable. The use of EA versus EAA as an outcome did not strongly impact estimates. Applying the optimal model in real-world data uncovered an accelerated EA rate in males and an advanced EA that decelerates over time in children with higher birthweight.

**Conclusion:**

Our results can serve as a guide for forthcoming longitudinal EA studies, aiding in methodological decisions that may determine whether an association is accurately estimated, overestimated, or potentially overlooked.

## Background

Chronological age, the passage of time since birth, does not fully capture an individual’s state or pace of biological aging [1]. Genetics, along with various environments, behaviours, and diseases faced throughout the life course appear to be potent causes of these disparities. While the exact biochemical mechanisms mediating these effects remain largely unknown, there is emerging evidence of a role for epigenetics, a biological process that can induce changes in gene expression without changing the underlying DNA sequence [2]. The adaptable nature of epigenetic modifications is therefore of utmost interest when evaluating the effects that exposures have on health and lifespan.

DNA methylation (DNAm) is the most studied, most stable, and easiest-to-measure epigenetic modification. DNAm most commonly occurs at cytosine nucleotides that are followed by guanine, known as CpG sites. A decade ago, Horvath [3] as well as Hannum and colleagues [4] introduced algorithms, also known as epigenetic clocks, that identified a set of CpG sites whose methylation state can be used to accurately estimate chronological age. The resulting measure is called epigenetic age (EA). Additional epigenetic clocks have since been published; some aim to best estimate chronological age [5], while others focus on health and mortality [6–11]. Over the past decade, the scientific community has thoroughly investigated EA-related associations, largely indicating relations between advanced EA and adverse health outcomes [12–15].

In most of those studies, EA was investigated in a cross-sectional setting. However, the increasing accessibility of longitudinal epigenetic cohort data [16] has created growing interest in studying EA over time [17–45]. Unfortunately, there are many disparities in modelling strategies across these studies. Our study investigated whether these disparities impacted findings to an extent that might lead to false conclusions, through significantly inflating effects or leaving true associations unnoticed. We evaluated the robustness of methods using simulations. To test our results in real-world data, we applied the same methods in two examples from the Avon Longitudinal Study of Parents and Children (ALSPAC) [46,47] involving sex and birthweight as predictors of longitudinal EA. Our aim was to provide readers with practical guidance in modelling choices that fit their data and maximise insights in epidemiological relations.

## Methods

### Modelling Longitudinal Epigenetic Age

Numerous approaches exist to model effects that exposures have on longitudinal outcomes like EA. Approaches differ in (i) the choice of model, (ii) outcome, and (iii) time variables, as well as (iv) the number of repeated measures included in those methods.

The three most frequently applied models are: linear mixed effect models (LME) [17–21,24,26,31,32,34–37,39,42], generalized estimating equations (GEE) [22,27,30,44], and Δ aging [23,28,38,40,41,43,56,57]. LME models and GEE both analyse repeated measures, like tracking changes in EA over time in an individual’s life, and model differences in mean trends between groups, such as those exposed to a factor compared to those who were not. In both methods, the fixed effect (*β*_2_), which stays consistent throughout all measures, as well as the interaction effect (*β*_4_), which accumulates over time, are typically modelled as:

Formula 1 $${\text{Outcome}}_{\text{ij}}=\beta_{0\text{i}}+\beta_{1}{\text{Time}\;\text{Variable}}_{\text{ij}}+\beta_{2}{\text{Exposure}}_{i}+\beta_{3}{\text{Sex}}_{i}+\beta_{4}\left({\text{Time}\;\text{Variable}}_{\text{ij}}\times {\text{Exposure}}_{i}\right)+\epsilon_{ij}$$

Where *Outcome*_*ij*_ is either EA or Epigenetic Age Acceleration (EAA, the residual resulting from regressing EA on chronological age) measured in individual *i*, at timepoint *j*. To accommodate longitudinal changes, both models account for time. Time variables commonly used, summarized in Figure 2, range from chronological age at the time of measurement to age-independent variables, including the duration in days or years between measurements, numerical ranks (e.g., 1, 2, 3) or factorized values (e.g., F07, F09, F15).

Another method to analyse variations in temporal changes is a two-step approach involving “Δ aging” (“delta aging”). This method is limited to studies with two repeated measures, as it quantifies the difference between measurements, often referred to as the “Δ aging” score, and then compares these scores between groups, for example using linear regression. Firstly, Δ aging is typically calculated as the difference between a follow-up and a baseline measure of either EA or EAA, with or without adjustment for the duration of time between measurements:

Formula 2 $$\Delta \;\text{Aging}={\text{Outcome}}_{2}-{\text{Outcome}}_{1}\;\Delta \;\text{Aging}(\text{age}\;\text{adjusted})=\frac{{\text{Outcome}}_{2}-{\text{Outcome}}_{1}}{{\text{Age}}_{2}-{\text{Age}}_{1}}$$

Where *Outcome*_1_ and *Age*_1_ represent EAA or EA and age at the initial measure, while *Outcome*_2_ and *Age*_2_ correspond to the follow-up measure. Secondly, models such as linear regression can be used to compare trends in Δ aging between the different groups:

Formula 3 $$\Delta \;{\text{Aging}}_{\text{i}}=\beta_{0}+\beta_{1}{\text{Exposure}}_{i}+\beta_{2}Sex_{i}+\epsilon_{i}$$

Where *Δ Aging*_i_ is the above-described difference score between two measures for individual *i*.

### Study Population

This study used longitudinal DNAm data generated as part of the Avon Longitudinal Study of Parents and Children (ALSPAC) [46,47]. Initially, ALSPAC recruited 14,541 pregnant women, resident in Avon, UK with expected dates of delivery between April 1991 and December 1992. Of the initial 14,541 pregnancies, 14,062 resulted in live births and 13,988 children were alive at 1 year of age. To bolster the initial sample, 1,000 additional children were included after the initial participants were approximately 7 years old, increasing the total sample of data collected after age 7 to 14,901. As part of the Accessible Resource for Integrated Epigenomic Studies (ARIES) [16], a sub-sample of 1,018 mother-child pairs have undergone genome-wide DNAm analysis. Here, we included up to three within-person DNA methylation measures, drawn from blood, at age 7, 9, and 15–17. DNAm wet-lab and pre-processing analyses were performed at the University of Bristol as part of the ARIES project [16,58].

### Simulation Study

**Figure 1:**
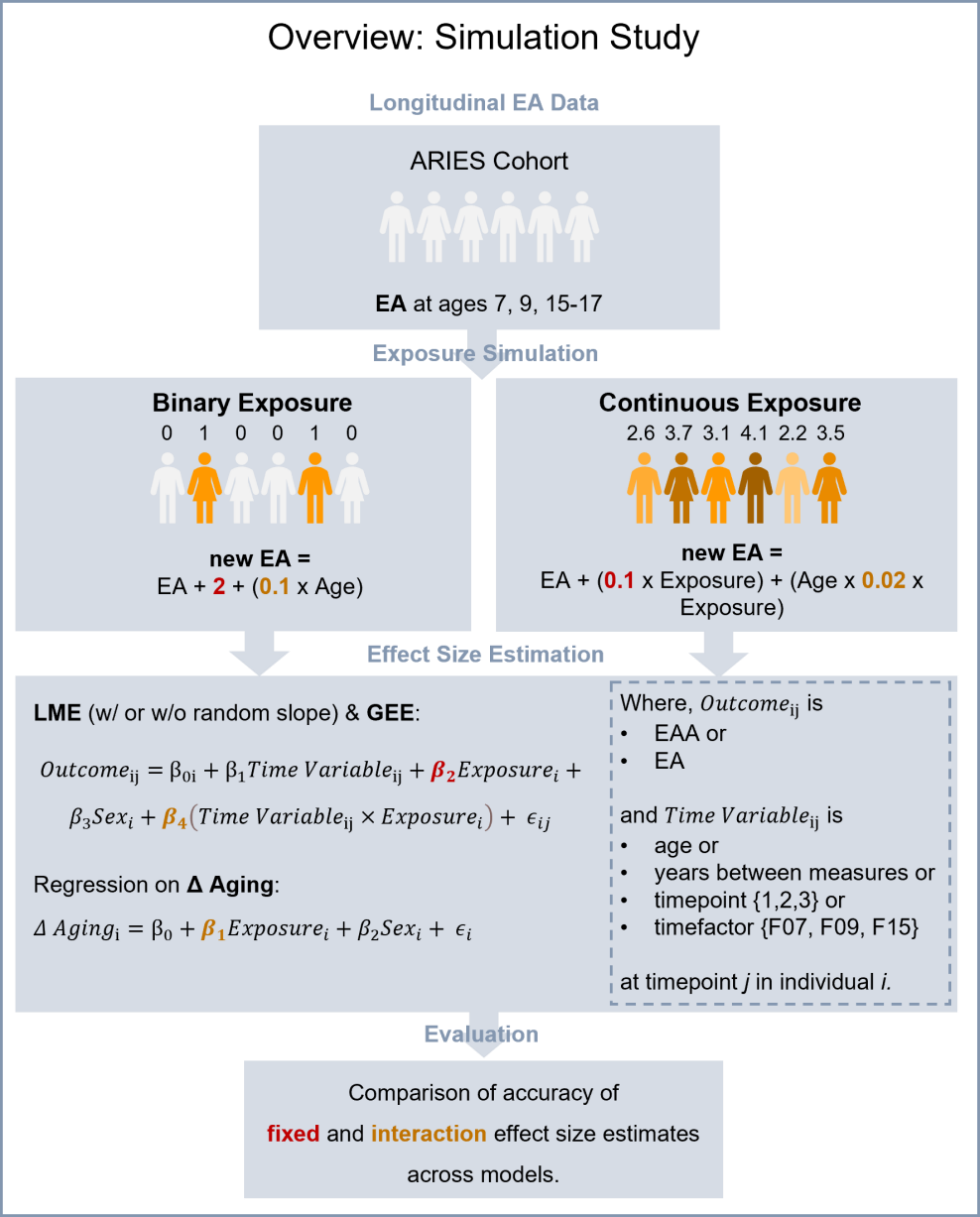
Overview of simulation study. Simulations were based on longitudinal ARIES cohort data [16] available at ages 7, 9, and 15 to 17. Epigenetic Age (EA) was calculated using the Horvath clock [3] or GrimAge2 [10] in separate simulations. The original EA measure was then altered based on a simulated exposure. In each binary exposure simulation, a random n=100 individuals had their original EA increased by 2 years (fixed effect), which accumulated by 0.1 year of EA per year of life (interaction effect). In each continuous exposure, all individuals were assigned a value (N(3.5, 0.5^2^)) which impacted their original EA by 0.1 years, times the level of exposure (fixed effect), and caused an interaction between the exposure and age by 0.02 (interaction effect). In the next step of our simulation, a series of methods was applied to model the simulated effects. Models are Linear Mixed Effect Models (LME), Generalized Estimating Equations (GEE), regression on difference between two Epigenetic Age (EA) measures (Δ aging). Outcome variables included are Epigenetic Age Acceleration (EAA, residual from regressing EA on age), or EA itself. We ran n = 1000 simulations for each exposure type (binary vs. continuous), epigenetic clock (Horvath vs. GrimAge2), and different scopes of data (two measures: Age 7 and 15–17 vs. three measures: Age 7, 9, and 15–17).

**Figure 2:**
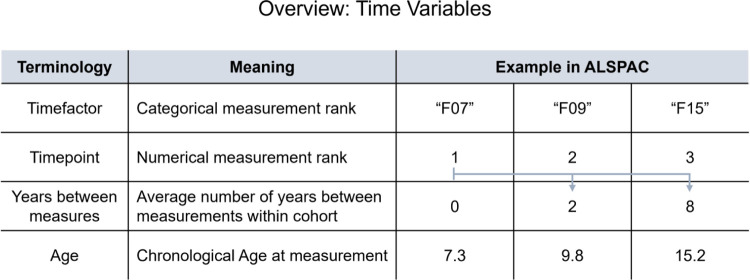
Overview of common time variables included in longitudinal epigenetic age studies and their application in ARIES cohort data. Age variables in our example should be interpreted as a single study participant’s age at measurement, since chronological age differs between individuals.

To better understand the extent to which methodological choices influence the robustness of results, we compared commonly used methods (introduced under *Modelling Longitudinal Epigenetic Age*). We conducted a series of simulations (n = 1,000), in which we manipulated longitudinal EA data from the ALSPAC cohort, applied all models introduced above, and compared the accuracy of effect estimates across methods (Figure 1).

Simulations were based on longitudinal DNAm data from ARIES [16], condensed into EA measures. To compare EA derived from conceptually different epigenetic clocks, we investigated EA calculations from both the Horvath clock [3] and GrimAge2 [10] in separate simulation cycles. In our binary exposure simulations, 100 participants were randomly selected as “exposed” (n = 918 “unexposed”). To create an association between the exposure and the average outcome, EA was increased by a fixed effect of 2 years in those 100 “exposed” individuals (*β*_2_). Additionally, to create an association between the exposure and change in the average outcome over time, an interaction with chronological age by 0.1 years EA per year of age was included (*β*_4_). In our continuous exposure simulations, all participants were randomly assigned a value ((3.5, 0.5^2^)), which affected EA with a fixed effect coefficient of 0.1 (*β*_2_) and an interaction coefficient of 0.02 (*β*_4_).

We then iterated (n = 1,000) through all (i) models, (ii) outcomes and (iii) time variables discussed above, including (iv) different numbers of repeated measures (two timepoints vs. three timepoints). First, we evaluated three models (i): LME models (with and without random slope term), GEE, and regression on Δ aging. Second, we assessed two outcomes within these models (ii): EA an EAA. Third, we investigated four time variables (iii): age, years between measures, numerical ranks {1, 2, 3}, and factorized values {F07, F09, F15}. Δ aging was calculated with and without adjusting for the time between initial and follow-up measure. Fourth, we evaluated the impact of repeated measures (iv) on model accuracy by fitting LME models and GEE with two or three timepoints. Δ aging was limited to only two timepoints.

To compare robustness across methods and variables, we extracted fixed (*β*_2_) and interaction (*β*_4_) effect estimates from all models and evaluated how accurate they met the simulated exposure effect. We measured each model’s performance by comparing whether the 95% confidence interval (CI) contained the simulated effect size. Models resulting in CI that were fully above or below the simulated effect were labelled as “inflated” or “deflated”, while models resulting in CI that contained the simulated effect were defined as performed best.

### Real-world example

To apply our simulation results in a real-world example, we used sex and birthweight as accessible biological parameters and examples of binary and continuous predictors of longitudinal EA. Longitudinal EAA or EA at ages 7 and 15–17, derived from the Horvath clock [3] and GrimAge2 [10], were modelled as the outcome. We applied all models and time variables discussed above and included sex and cell type proportions as covariates. Cell counts were estimated using the Houseman algorithm [59] applied to ALSPAC DNAm data with a peripheral blood reference [60].

In LME models and GEE, the fixed (*β*_2_) and interactive (Formula 4: *β*_9_, Formula 5: *β*_10_) effects were estimated as:

Binary exposure (Biological Sex):

Formula 4 $${\text{Outcome}}_{ij}=\beta_{0i}+\beta_{1}{\text{Time}\;\text{Variable}}_{ij}+\beta_{2}{\text{Sex}}_{i}+\beta_{3}NK_{ij}+\beta_{4}{\text{Granu}}_{ij}+\beta_{5}{\text{Mono}}_{ij}+\beta_{6}CD4T_{ij}+\beta_{7}CD87_{ij}+\beta_{8}{\text{Bcell}}_{ij}+\beta_{9}\left({\text{Time}\;\text{Variable}}_{ij}\times {\text{Sex}}_{i}\right)+\epsilon_{ij}$$

Continuous exposure (Birthweight):

Formula 5 $${\text{Outcome}}_{ij}=\beta_{0i}+\beta_{1}{\text{Time}\;\text{Variable}}_{ij}+\beta_{2}{\text{Birthweight}}_{i}+\beta_{3}{\text{Sex}}_{i}+\beta_{4}NK_{ij}+\beta_{5}{\text{Granu}}_{ij}+\beta_{6}{\text{Mono}}_{ij}+\beta_{7}CD4T_{ij}+\beta_{8}CD8T_{ij}+\beta_{9}{\text{Bcell}}_{ij}+\beta_{10}\left({\text{Time}\;\text{Variable}}_{ij}\times {\text{Birthweight}}_{i}\right)+\epsilon_{ij}$$

Where *Outcome*_*ij*_ is EA or EAA, measured in individual *i*, at timepoint *j*.

Different trends (*β*_1_) in Δ aging (with and without adjusting for time between measures) across groups were modelled using linear regression:

Binary exposure (Biological Sex):

Formula 6 $$\Delta \;\text{Agin}g_{\text{i}}=\beta_{0}+\beta_{1}Sex_{i}+\epsilon_{i}$$

Continuous exposure (Birthweight):

Formula 7 $$\Delta \;{\text{Aging}}_{\text{i}}=\beta_{0}+\beta_{1}{\text{Birthweight}}_{i}+\beta_{2}{\text{Sex}}_{i}+\epsilon_{i}$$

Where *Δ Aging*_i_ is the difference score between two measures for individual *i*.

## Results

### Simulation Study

**Figure 3:**
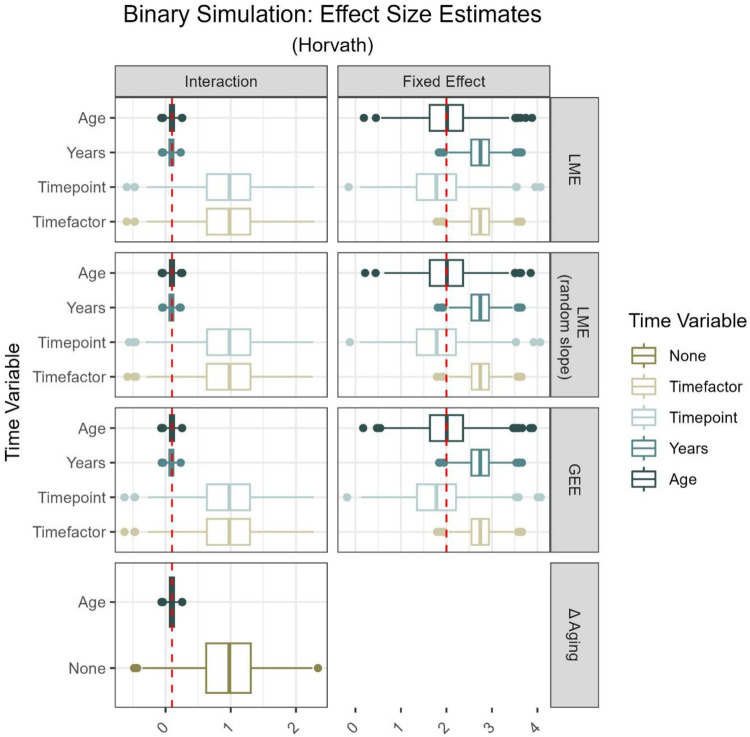
Summary of n = 1,000 simulations from ARIES data (two measurements, age 7 and 15 or 17) [16]. Rows show the distribution (median, 25^th^ and 75^th^ percentile, minimum, maximum and outliers) of effect size estimates derived from different models and time variables included in those models respectively. The two columns differentiate between estimates of the interaction term as well as the fixed effect. Simulated effect sizes are marked in red (interaction=0.1; fixed effect=2.0). Time variables are chronological age (Age), years between measures (Years), number of measure (Timepoint, i.e., 1, 2, 3), factorized measure (Timefactor, i.e., F07, F09, F15). Models are Linear Mixed Effect models (LME), Generalized Estimating Equations (GEE), and regression on difference between two epigenetic age (EA) measures (Δ aging). All models contained Horvath clock derived EA as outcome [3].

Figure 3 and **Tables and *Figures***

Table 1 summarize the effect estimates for a binary exposure for all models and time variables considered, using a subset of two within-person EA measures derived from the Horvath clock. Additional tables, summarizing the complete range of effect estimates, including the continuous exposure and models involving three within-person measures of EA derived from the Horvath clock as well as GrimAge2, can be found in the supplemental material (**Supplement Figure 1–16** & Table 1–**4**). Across all approaches, the choice of time variable had the most substantial impact on the effect estimate bias, which stayed consistent across models (LME models, GEE and Δ aging) and outcome variables (EA, EAA). Including chronological age in the model gave unbiased accurate estimates, while other age-independent time variables led to inflated results.

For both fixed and interaction effects alike, we obtained similar estimates across LME models and GEE, holding the time variable constant. Models that included timepoint as their time variable resulted in a slightly higher proportion of deflated fixed effect estimates than models including age, when using two within-person measures (LME model with random slope, binary simulation: timepoint 5% deflated, age 2% deflated; **Supplement Figure 5** and **7**). The majority of estimates from models accounting for years between measures or categorical time overestimated the simulated fixed effect (LME model with random slope, binary simulation: 80% inflated; **Supplemental Figure 5** and **7**). When including three repeated within-person measures in the analysis, all estimates from models incorporating time as a categorical variable were inflated.

Regression on Δ aging showed high precision and accuracy for estimating the interaction coefficients, when adjusted for years between measures. Similar performance in approximating the age-exposure interaction was achieved using LME models and GEE accounting for time through either chronological age or years between measures. Including either categorical time or numerical timepoint led to overestimated effects across all models, resulting in up to 52% inflated interaction estimates (LME model with random slope, binary exposure: timepoint, **Supplemental Figure 5** and **7**). Models based on three repeated within-person measures resulted in exclusively deflated interaction estimates, when including categorical time as the time variable.

### Real-world Examples

**Supplemental Table 5** and **6** as well as **Supplemental Figures 17–20** show the complete range of effect size estimates for both exposures on longitudinal EA, across models, clocks, and time variables. Again, the choice of time variable had the most substantial impact on effect estimates, which stayed consistent across models and outcome variables.

**Figure 4:**
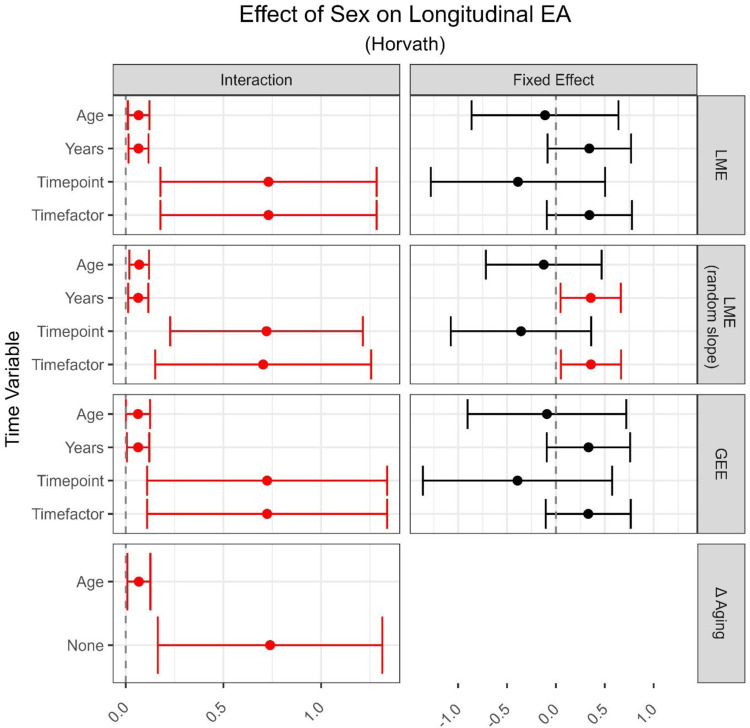
Application results on the effect of sex on EA over time. Included were two within-person measures (age 7 and 15 or 17) from the ARIES cohort [16]. Rows contain effect size point estimates as well as 95% confidence intervals derived from different models and time variables included in those models respectively. Effect estimates are based on females as reference. Significant estimates are marked in red (p < 0.05). The two columns differentiate between estimates of the interaction term as well as the fixed effect. Models are Linear Mixed Effect models (LME), Generalized Estimating Equations (GEE), and regression on difference between two epigenetic age (EA) measures (Δ aging). Time variables are chronological age (Age), years between measures (Years), number of measure (Timepoint, i.e., 1, 2, 3), factorized measure (Timefactor, i.e., F07, F09, F15). All models contained Horvath clock derived EA as outcome [3] and were corrected for cell type proportion.

The right column in Figure 4 compares the fixed effect estimates of biological sex on longitudinal EA between the ages 7 and 15–17 years across methods. Different model and outcome choices led to very similar estimates of fixed effects, so we compared the choice of time variable within the LME model (random slope) using Horvath EA as the outcome. Males had 0.12 years lower average EA compared to females (95% CI −0.71, 0.48) using the chronological age as the time variable, and based on our simulation study, this is an unbiased estimate. With this result as a reference, using either years-between-measures or categorical time resulted in estimates three times larger and in the opposite direction (males were on average 0.36 [95% CI 0.05, 0.66] years older). The use of timepoint led to estimates almost three times larger in the same direction (males were on average 0.34 [95% CI −1.06, 0.37] years younger). While point estimates differed using GrimAge2 (likely due to the different training outcome measures of these clocks), the choice of time variable had a similar effect as seen when using the Horvath clock. In both Horvath and GrimAge2 EA measure-based models, including age or timepoint as a time variable led to much wider confidence intervals compared to using categorical time or time-between-measures.

Interaction effects between sex and age, leading to an accumulating positive or negative effect on EA over time are shown in the left column of Figure 4. The choice of model or outcome led to approximately equal estimates holding the time variable constant. Once again, while effect sizes differed profoundly between clocks, the effect of different time variables was similar using Horvath or GrimAge2. Using LME models with age as the time variable, male EA increased by an extra 0.06 years per year of age, compared to females (95% CI 0.01, 0.12). Using either years as the time variable, or Δ aging, led to the same point estimate. However, using either timepoint or categorical time inflated the interaction effect drastically (0.72, 95% CI 0.16, 1.27).

**Figure 5:**
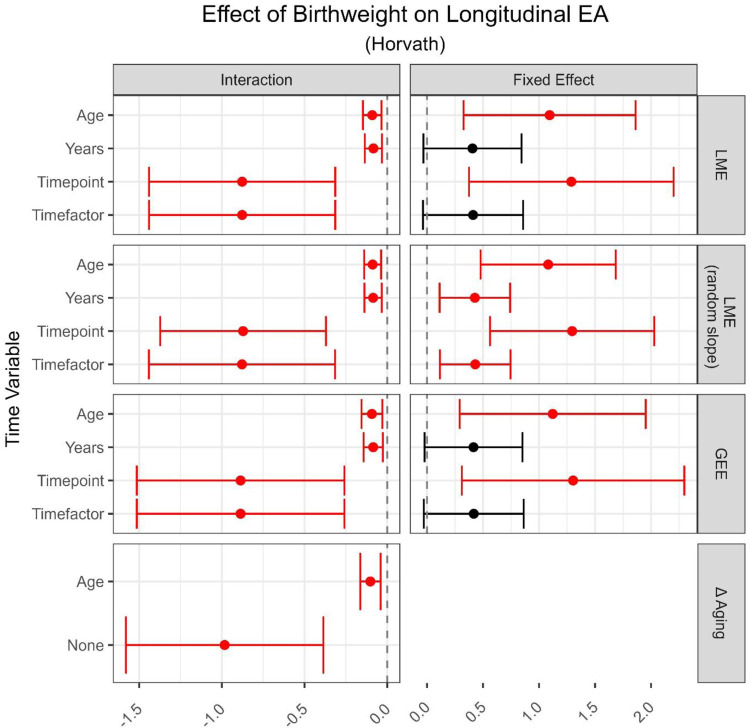
Application results on the effect of birthweight on EA over time. Included were two within-person measures (age 7 and 15 or 17) from the ARIES cohort [16]. Rows contain effect size point estimates as well as 95% confidence intervals derived from different models and time variables included in those models respectively. Significant estimates are marked in red (p < 0.05). The two columns differentiate between estimates of the interaction term as well as the fixed effect. Time variables are chronological age (Age), years between measures (Years), number of measure (Timepoint, i.e., 1, 2, 3), factorized measure (Timefactor, i.e., F07, F09, F15). Models are Linear Mixed Effect models (LME), Generalized Estimating Equations (GEE), and regression on difference between two epigenetic age (EA) measures (Δ aging). All models contained Horvath clock derived EA as outcome [3] and were corrected for sex and cell type proportion.

The fixed effect estimates of birthweight on longitudinal EA between the age 7 and 15–17 years of age are shown in the right column of Figure 5 and in **Supplemental Table 5**. Due to similar estimates across models and outcome choices, we again compared results for different time variables within LME models (random slope) using Horvath EA as the outcome. Using chronological age as the time variable showed that an increase in birthweight of 1kg is associated on average with an additional 1.08 years of EA (95% CI: 0.48, 1.69). Using this estimate as a reference (assuming it is unbiased as per our simulation results), then using either years-between-measures or categorical time as the time variable led to an estimate 2.5 times smaller (0.43 [95% CI 0.11, 0.75] years average increase per 1 kg). The use of timepoint increased estimates by 25% (1.3 [95% CI 0.56, 2.03] years average increase per kg). Models using GrimAge2 measures led to opposite effect estimates, none of which showed statistical significance. Trends across time variables were similar between Horvath and GrimAge2 EA measure-based models, leading to much wider confidence intervals when including age or timepoint as time variable compared to using categorical time or time-between-measures.

Estimates of interaction effects between birthweight and age are shown in the left column of Figure 5 and in **Supplementary Table 6**. Again, the selection of either the model or the outcome led to similar estimates when keeping the time variable constant. Using LME models with age as the time variable, EA decreased on average by 0.09 years per year of age for each kg increase in birthweight (95% CI −0.14, −0.04). Using years-between-measures, or age-adjusted Δ aging, as the time variable led to a similar point estimate. Regressing on non-age-adjusted Δ aging or including timepoint or categorical time in the model inflated the interaction effect by a factor of 10 (−0.87, 95% CI −1.44, −0.32).

## Discussion

The findings of this study present valuable insight into the intricacies of modelling associations with longitudinal epigenetic age, emphasizing the critical influence of different exposure and time variables on the robustness of effect estimates and conclusions.

### Simulation & Real-world Examples

All simulation and real-word analyses showed consistent estimates across models and outcome variables, while the choice of time variable significantly impacted the accuracy and precision of effect estimates. In simulations, including chronological age as the time variable in an LME model or GEE led to the highest number of correct fixed effect and interaction effect estimates. Hence, models accounting for chronological age also produced the most robust results in our real-world analysis of biological sex and birthweight. When estimating interaction effects, i.e. the accumulating effect that an exposure has on the pace of longitudinal EA, models including timepoint or categorical time had the highest number of incorrect effect size estimates (inflated or deflated) in simulations. We observed a difference of similar magnitude in interaction effect estimates in both real-world analyses compared to estimates from models using chronological age. Including years-between-measures or categorical time led to 80% inflated fixed effect estimates and narrow confidence intervals in our binary exposure simulation. This bias led to what appears like a false positive finding in the real-world analysis of sex.

### Application Examples: Epidemiological Conclusion

The two application examples not only lend support to our simulation findings but also yield several novel epidemiological findings. First, our study suggests that males have a higher rate of GrimAge2 epigenetic aging than females. More specifically, males showed an extra 0.07 years (95% CI: 0.02, 0.12) EA increase per year of life on average, compared to females. Although cross-sectional studies across different age groups, ancestries, and epigenetic clocks have identified an association between sex and EA [35,48,49], no prior study, to our knowledge, has shown that this effect accumulates longitudinally. Those longitudinal fixed effect differences were specific to GrimAge2 and were not detected using EA estimates from Horvath’s clock, which is likely caused by conceptual differences between these clocks; the Horvath clock is trained to best predict chronological age while GrimAge2 was trained to predict health- and lifespan. Biological aging studies focusing on other molecular biomarkers to investigate sex-specific differences support the slower rate of aging in women [50,51].

Second, our results suggest a positive association between birthweight and longitudinal EA based on Horvath EA measures (1.08 additional years of EA per increase in kg birthweight, 95% CI: 0.48, 1.69), as well as a negative birthweight/age interaction effect over time (−0.09 years decrease of EA per year per increase in kg birthweight, 95% CI: −0.14, −0.04). These results indicate that children born with higher birthweight have higher EA on average, while their pace of EA appears to slow down over the period measured, compared to children born with lower birthweight. Several other studies have tested associations between birthweight and EA, with the overall consensus that lower birthweight predicts higher EA [52–55]. However, Simpkin et al. (2016) found that birthweight was positively associated with EA at age 7, but negatively associated at age 17. Most studies analysed data cross-sectionally, included dichotomized birthweight rather than continuous, and were based on EA measures in adult cohorts. Furthermore, associations were predominantly identified in males [53,54] or in male-dominated cohorts [52]. Future large-scale studies are needed to clarify longitudinal relationships and explore the effect birthweight has on EA throughout the life course.

### Recommendations for Future Studies

As we recognize the importance of methodological choices, this section offers recommendations and guidance for researchers embarking on similar investigative paths. We highly recommend the use of LME models or GEE, including chronological age as the time variable, for studies working with repeated EA measures. These approaches improve precision and accuracy of fixed and interaction effect estimates. Alternatively, research evaluating interaction effects based on only two within-person measures can yield similar validity by using linear regression on age-adjusted Δ aging. We acknowledge that due to limited data collection or access, it is not always possible to implement with best-possible model. In cases where chronological age is not accessible and the cohort under study was measured in synchronized waves, we recommend the use of numerical timepoint to get more accurate fixed effect estimates. The effect size might appear slightly attenuated compared to models including age but is less susceptible to false positives. In studies aimed at exploring interaction effects, it is advisable to opt for years between measures instead of timepoint in the absence of chronological age. Factorized categorical time should be avoided due to its potential to introduce bias in fixed effect and interaction effect estimates, especially in studies incorporating more than two within-person measurements.

### Strengths and Limitations

The main strength of this study lies in its comprehensive evaluation of longitudinal EA models, thereby offering valuable insight to direct future research towards more reliable results. The increasing accessibility of repeated DNAm measures and growing interest in comprehending the effects of exposures on EA and its subsequent influence on health have shown the necessity for guidelines to address this issue robustly. A further strength of this study is the incorporation of both simulation and application in two real-world examples, which support the credibility and applicability of our results. One limitation worth noting is that the real-world examples in this study were modelled using only two EA measures, which might have impacted the comprehensiveness of our epidemiological findings. However, our simulation based on three repeated EA measures suggests results from models including age as time variable are consistent across different scopes of data. Second, we limited our methodological evaluation to common models and time variables found in recent literature [17–45] and excluded rare and simplistic approaches. While there are certainly various other methods available for this purpose, we assume that our study has addressed the applications most pertinent to most epidemiological studies.

## Conclusions

In conclusion, our study presents a comprehensive evaluation of various methods utilized in modelling exposure effects on EA over time. Through a combination of simulation and real-world analyses, we have demonstrated that the methodological decisions made in longitudinal EA modelling significantly impact the reliability of effect estimates. Our findings highlight that LME models or GEE, using chronological age as the time variable, are the optimal approach. Moreover, recognizing the constraints faced by some studies regarding data availability, we have provided practical recommendations to accommodate such limitations. This thorough assessment serves as a valuable resource for guiding future epidemiological epigenetic aging research endeavours. By optimizing methodological approaches based on the insights from our study, researchers can enhance the depth and accuracy of their investigations, ultimately advancing our understanding of the complex interplay between exposures and epigenetic aging processes.

## Figures and Tables

**Table 1:** Summary of n = 1,000 simulations from ARIES data (two measurements, age 7 and 15 or 17) [16]. The effect size of the simulated binary exposure was 2.0, with an interaction effect of 0.1. Average fixed effect and interaction estimates and 95% confidence intervals (CI) from all models are displayed as columns, as well as the average bias from the simulated effect. Rows represent time variables included in the respective model. Models are Linear Mixed Effect models (LME), Generalized Estimating Equations (GEE), and regression on difference between two epigenetic age (EA) measures (Δ aging). Time variables are chronological age (Age), years between measures (Years), number of measure (Timepoint, i.e., 1, 2, 3), factorized measure (Timefactor, i.e., F07, F09, F15). All models contained Horvath clock derived EA as outcome [3].

Simulated Effect Size:		0.1	2.0
Model	Time Variable	Interaction Effect Estimate (95% CI)	Fixed Effect Estimate (95% CI)
LME	Age	0.1 (0,0.19)	2.01 (0.72,3.3)
	Years	0.09 (0,0.18)	2.75 (2.02,3.47)
	Timepoint	0.96 (0.02,1.91)	1.78 (0.25,3.31)
	Timefactor	0.96 (0.02,1.91)	2.75 (2.01,3.49)
LME (random slope)	Age	0.1 (0.01,0.19)	2.01 (0.99,3.03)
	Years	0.09 (0,0.18)	2.75 (2.22,3.27)
	Timepoint	0.97 (0.12,1.81)	1.78 (0.55,3.01)
	Timefactor	0.96 (0.02,1.91)	2.75 (2.22,3.27)
GEE	Age	0.1 (−0.01,0.21)	2.01 (0.86,3.16)
	Years	0.09 (−0.01,0.19)	2.75 (2.23,3.27)
	Timepoint	0.96 (−0.08,2.01)	1.78 (0.4,3.17)
	Timefactor	0.96 (−0.08,2.01)	2.75 (2.23,3.27)
Δ aging	Age	0.1 (0,0.2)	
	None	0.97 (0.01,1.93)	

## Data Availability

ALSPAC data is available on request at http://www.bristol.ac.uk/alspac/researchers/access/. Details of all the data is available through a fully searchable data dictionary and variable search tool on the ALSPAC study website:http://www.bristol.ac.uk/alspac/researchers/our-data/.
